# Transcriptomic and Widely Targeted Metabolomic Approach Identified Diverse Group of Bioactive Compounds, Antiradical Activities, and Their Associated Genes in Six Sugarcane Varieties

**DOI:** 10.3390/antiox11071319

**Published:** 2022-07-04

**Authors:** Muhammad Junaid Rao, Mingzheng Duan, Jihong Wang, Shijian Han, Li Ma, Xinyi Mo, Min Li, Lihua Hu, Lingqiang Wang

**Affiliations:** 1 Guangxi Key Laboratory of Sugarcane Biology, College of Agriculture, Guangxi University, 100 Daxue Rd., Nanning 530004, China; mjunaidrao@webmail.hzau.edu.cn (M.J.R.); duanmingzheng@gxu.edu.cn (M.D.); hsjly@gxu.edu.cn (S.H.); 2017301029@st.gxu.edu.cn (L.M.); 2017301030@st.gxu.edu.cn (X.M.); 1517402001@st.gxu.edu.cn (M.L.); 2 State Key Laboratory for Conservation and Utilization of Subtropical Agro-Bioresources, College of Agriculture, Guangxi University, 100 Daxue Rd., Nanning 530004, China; 3 Department of Life Science, Tangshan Normal University, Tangshan 063000, China; wjhongok@tstc.edu.cn

**Keywords:** LC-MS/MS, sugarcane rind, antioxidant activity, amino acid, *Arabidopsis*

## Abstract

Sugarcane is cultivated mainly for its high sucrose content but it can also produce many metabolites with promising antioxidant potential. However, very few studies have been reported on the biosynthesis of metabolites in sugarcane to date. In this study, we have identified a wide range of amino acids and organic acids in the rind of six sugarcane varieties by the LC-MS/MS method. A total number of 72 amino acids and 55 organic acid compounds were characterized; among these, 100 were reported for the first time. Moreover, 13 amino acids and seven organic acids were abundantly distributed in all varieties tested and considered major amino acids and organic acids in sugarcane. The variety Taitang134 (F134) showed the highest content of total amino acids, whereas the varieties ROC16 and Yuetang93/159 (YT93/159) had maximum content of organic acids. The amino acids of the rind extract presented higher antioxidant capacity than the organic acids of the rind extract. In addition, the transcriptomic and metabolic integrated analysis highlighted some candidate genes associated with amino acid biosynthesis in sugarcane. We selected a transcription factor gene, *MYB*(*t*), and over-expressed it in *Arabidopsis*. The transgenic plants showed a higher accumulation of amino acids with higher antiradical activity compared with the wild-type *Arabidopsis* plants. Thus, we characterize a wide range of amino acids and organic acids and their antiradical activities in different sugarcane varieties and present candidate genes that can be potentially valuable for the genetic improvement of metabolites in sugarcane bagasse

## 1. Introduction

Sugarcane is the fifth largest crop cultivated in tropical and sub-tropical regions of the world, mainly in Brazil, China, India, and Mexico [1]. As a perennial crop, sugarcane encounters different abiotic and biotic stresses during its whole growth life. Consequently, sugarcane produces several bioactive compounds such as amino acid, organic acid, phenolic acid, flavonoids, anthocyanins, and proanthocyanidins to cope with these unfavorable environmental conditions [2,3]. The sugarcane rind plays a key role in protecting the stem sugar and produces many metabolites such as amino acids, organic acid alkaloids, phenolics, flavonoids, triterpenoids, etc., having strong antioxidants, antimicrobial, antibacterial, and antifungal activities. Some of these metabolic compounds, such as ferulic acid, gallic acid, and *p*-coumaric acid [4,5], have pharmacological and cosmetic values, others even have health-promoting effects on humans to improve HDL-cholesterol and lower cholesterol level in the blood [1,6]. However, few studies have been reported about the presence of these compounds in sugarcane juice and their quantification has not been accomplished in the cultivated sugarcane rind [4,7]. In particular, the phenolics and flavonoid compounds in sugarcane have been well studied and it is necessary to analyze the diversity and the relative bioactivity of the amino acids and organic acids in sugarcane.

Amino acids perform numerous functions in serving the building blocks of proteins, the precursors of primary and secondary metabolites with remarkable antioxidant activities, and providing tolerance to abiotic and biotic stress in plants [8,9,10,11]. In plants, methionine and cysteine can be transformed into glutathione through the redox cycle, to protect cellular organelles from unfavorable environmental conditions [12]. Lysine, arginine, and histidine produce strong antioxidant melanoidins by reacting with sugars [13]. The aromatic amino acids tryptophan, tyrosine, and phenylalanine share a resilient antioxidant activity that can inhibit the chain reaction of free radicals [14]. As for the synthesis of secondary metabolites [15], phenylalanine is converted into anthocyanins, flavonoids, isoflavonoids, tannins, and volatiles; tyrosine can derivate quinones, betalains, isoquinoline alkaloids, and especially half of the total lignins in grasses [16,17]; whereas tryptophan is converted into auxin, alkaloids, indole glucosinolates, and phytoalexins [18]. Thus, tryptophan, tyrosine, and phenylalanine can fix about thirty percent of photosynthetic carbon by producing thousands of metabolites for plant growth and development, defense, and environmental responses [19]. Therefore, it is important to identify the detailed pathway and associated genes in amino acid biosynthesis in sugarcane.

In the past two decades, several experiments have demonstrated that some organic acids perform various important functions in energy production, tolerance to environmental stress, formation of the precursors for amino-acid biosynthesis, and heavy metal tolerance. Organic acids, in particular, can help plants to cope with nutrient deficiencies and other challenging environmental conditions [11,20,21]. The organic acids are produced mainly in mitochondria via the Krebs cycle; some are produced via the glyoxylate cycle. Organic acids are found in small pools of mitochondria due to the catalytic nature of the Krebs cycle, eventually being stored in the vacuoles [20]. Among all living organisms, total organic acid contents are relatively highest in plant tissues [21]; something which may explain their essential role as photosynthetic intermediates. Besides this, organic acids have a key role in balancing cation excess and osmotic adjustment. However, the concentration, accumulation, and composition of organic acids change dramatically among species, varieties, plant tissues, and even at different developmental stages [21,22].

Until now, sugarcane metabolism has been less explored. Only a few studies have been reported about the identification of sugar, phenolics, flavonoids, and anthocyanin compounds in sugarcane [23], and no report has focused on the organic acid and amino acid compounds in cultivated sugarcane. In these cases, we performed a metabolomic analysis by widely targeted liquid chromatography–mass spectrometry/mass spectrometry (LC-MS/MS) in six different sugarcane varieties, to explore the diversity in contents of organic acid and amino acid compounds in the rinds of six different sugarcane varieties. Moreover, the antioxidant capacity of the total amino- and organic acid extracts were evaluated. Using the integrated metabolic profiling and RNA-seq analysis, we discovered the candidate genes involved in the biosynthesis of amino acid compounds. Finally, the function of one regulatory gene, *MYB*(*t*), was verified by ectopically overexpressing it in *Arabidopsis*. Our results will provide a basis for the subsequent gene function validation, molecular marker screening, and genetic improvement of sugarcane varieties in future.

## 2. Materials and Methods

Six cultivated sugarcane varieties including ROC22, YT93/159, F134, YT71/210, ROC16, and Taitang172 (F172) were used for amino acids and organic acid evaluation. The varieties were grown in the fields of the campus of Guangxi University, Nanning, China. The samples were collected from the rinds of six-month-old sugarcane with three biological repeats. Fresh rind samples for transcriptomic studies were frozen in liquid nitrogen and stored at −80 °C until use. The samples for metabolomic analysis were vacuum freeze-dried before the LC–MS/MS operation.

### 2.1. Transcriptomic and qPCR Analysis

Total RNA was extracted from fresh rind samples by using TRIzol RNA extraction kit (Invitrogen, Carlsbad, CA, USA). RNA extraction, assembly preparation, and sequencing libraries were prepared by Berry Hekang Biotech. (Beijing, China). Illumina (NEB, Ipswich, MA, USA) RNA Library Prep Kit (NEBNext UltraTM) was used for sequencing libraries preparation, the typical sequences index codes were added to each sample, per the company’s guidelines. From all data sets, low-quality sequence reads were eliminated and paired-end reads were produced by Illumina HiSeq 2500 platform. The *Saccharum spontaneum* reference genome (http://sugarcane.zhangjisenlab.cn/sgd/html/download.html; the Saccharum Genome Database platform, assessed on 17 October 2021, the gene IDs represented in raw transcriptomic data were corresponding to the *Saccharum spontaneum* reference genome Table S2) was assessed for mapping of the clean reads by using hisat2. The PRJNA824938 is the associate accession number to cite the deposited sequencing data of the sugarcane transcriptomic study (http://www.ncbi.nlm.nih.gov/sra/PRJNA824938; accessed on 22 April 2022). For further gene annotation analysis, the following databases were used: Kyoto Encyclopedia of Genes and Genomes Ortholog database (KO); Protein family (Pfam); Gene Ontology (GO); non-redundant nucleotide sequences, NCBI (Nt); Swiss-Prot (A protein sequence database), Clusters of Orthologous Groups of proteins (KOG/COG), and non-redundant protein sequences, NCBI (Nr). The cutadapt was used for filtering the adaptor sequences whereas the fastqc was used for the quality check of RNA-seq. data. The cuffdiff was used for differentially expressed genes (DEGs) and the significantly DEGs were determined by |Log2FoldChange| ≥ 1 with <0.05 *p*-value [24]. The DEGs GO enrichment analysis was achieved by Wallenius non-central hypergeometric (GOseq) R package [25]. KOBAS software was used for statistical enrichment analysis of DEGs in KEGG pathways [26]. PCA, hierarchical cluster analysis (HCA) and histogram were achieved by HCAPCA and FactoMineR (R package for multivariate analysis) in R language (https://www.r-project.org/; accessed on 22 April 2022) [27,28].

For qRT-PCR, complementary DNA (cDNA) was prepared by using 1 µL of total RNA, according to the manufacture’s recommendations (Vazyme kit, Nanjing, China). The qPCR master mix Kit (ChamQ Universal SYBR^®^, Vazyme, Nanjing, China) was used. For quantitative real-time PCR analyses, the C1000™ thermal cycler (CFX96™ Real-Time) system was used. An amount of 10 µL of the reaction mixture was used whereas actin was used for internal control. The qPCR primers details are shown in Table S3.

### 2.2. Chemical and Reagents for Metabolic Analysis

HPLC-grade methanol (MeOH) was obtained from Merck Company (Darmstadt, Germany). In all metabolic experiments, ultrapure (Millipore, Bradford, PA, USA) water was used. Standards were purchased from Sigma-Aldrich (St. Louis, MO, USA; http://www.sigmaaldrich.com/united-states.html; accessed on 8 January 2021). Formic acid and hydrochloric acid were purchased from Sigma-Aldrich (Burlington, MA, USA) and Ningxiang Xinyang Chemical Co., Ltd. (Ningxiang, China), respectively. A 1 mg/mL concentration of stock standard solutions (in 50% MeOH) were prepared and stored at 4 °C for further analysis, while 50% MeOH was used for the dilution of stock solutions.

### 2.3. Extraction of Sugarcane Rind Samples

For metabolic determination, multiple reaction monitoring (MRM) was accomplished by Metware Biotechnology Co., Ltd. (Wuhan, China). A mixer mill (MM 400, Retsch) was used to grind the sugarcane freeze-dried rind samples for 1.5 min at 30 Hz (with zirconia bead). An amount of 50 mg of dried sample was weighed followed by overnight extraction with 0.5 mL of extraction solution (having methanol/water/hydrochloric acid 500:500:1, *v*/*v*/*v*) at 4 °C. After extraction, all the samples were vigorously shaken for 5 min followed by 5 min ultrasound, and then centrifuged at 4 °C for 3 min, at 12,000× *g*. The rind residue were extracted twice by repeating the above steps and the supernatants collected in a new tube followed by filtration with 0.22 μm paper (Anpel) before mass spectrometry examination.

### 2.4. Chromatography Mass Spectrometry Acquisition Conditions

Data acquisition instrument system mainly includes Ultra Performance Liquid Chromatography (UPLC) (SHIMADZU Nexera X2, https://www.shimadzu.com.cn/; accessed on 5 December 2021) and tandem mass spectrometry (Applied Biosystems 4500 QTRAP, http://www.appliedbiosystems.com.cn/; accessed on 8 December 2021). The analytical conditions were as follows: solvent system, methanol (formic acid 0.1%); water (formic acid 0.1%); chromatographic column, Agilent SB-C18 (1.8 µm, 2.1 mm × 100 mm); gradient program, at 0 min 95:5 *v*/*v*, for 6 min 50:50 *v*/*v*, for 12 min 5:95 *v*/*v* followed by 2 min hold, 14 min at 95:5 *v*/*v*, again 2 min hold; 0.35 mL/min of flow rate, 40 °C column temperature; and 4 μL of sample injection volume. At ESI triple quadrupole-linear ion trap (QTRAP)-MS effluent was connected.

### 2.5. Mass Spectrometry Conditions and Metabolic Analysis

Linear ion trap (LIT) and triple quadrupole scans (QQQ) were attained by QQQ-LIT mass spectrometer (Q TRAP), AB4500 Q TRAP ultra-performance liquid chromatography-mass spectrometry UPLC-MS/MS system, operating in positive and negative, two ion mode, with an electrospray ionization (ESI) Turbo ion spray (IS) crossing point, governed by (AB Sciex) Analyst 1.6.3 (Software version 1.6.3 HotFix 4; Shanghai, China). The parameters of ESI source operation were as follows: turbo spray; source temperature 550 °C; ion source gas (GSI), gas (GSII), and curtain gas were adjusted at 50, 60, and 25 psi, correspondingly; IS voltage 5500 V (+ion mode)/IS −4500 V (ion mode), collision induced ionization parameter is set to high. In QQQ and LIT mode, 10 and 100 jmol/L polypropylene glycol solution was used, respectively, for tuning and quality calibration instrument. QQQ scan using MRM mode and collision gas (ammonia) was set to medium. Through further optimal compound-specific de-clustering potential (DP) and collision energy (CE) optimization, the MRM ion pair of DP and CE were completed. Elution of metabolites, according to each period in every period monitoring a specific set of MRM ion pairs.

The metabolites in the samples were analyzed by qualitative and quantitative mass spectrometry based on MetWare database (MWDB) and MRM. Metabolite identification is based on the precise quality of metabolites, secondary fragments (MS2), isotope distribution and retention time (RT) of MS2 fragments. Through the intelligent secondary spectrum matching method independently developed by the company, the secondary spectrum and RT of metabolites in project samples are matched intelligently one by one with the secondary spectrum and RT of the MWDB database. MS and MS2 tolerances were set to 10 PPM and 20 PPM, respectively. Internal standard and quality control information is represented in the supplementary file. The raw metabolic data is represented in Table S3.

### 2.6. Agrobacterium Mediated Transformation and Phylogenetic Analysis

Columbia-0 wild-type *Arabidopsis* seeds were used for *MYB*(*t*) gene overexpression. The *Arabidopsis* seeds were firstly sown in petri-plates having Murashige and Skoog (MS) medium (25 g of sucrose per liter, 4.43 g of MS-dried medium photo technology laboratories, 5.7 pH, 10 g of agar) and then transferred to soil in the growth chamber (at 20–22 °C, 70% relative humidity, and 5000 LUX light intensity). The sugarcane complementary DNA was used to clone the *MYB*(*t*) gene (coding sequence) and intervened with the pK7WG2D binary gateway vector (Gateway technology, Invitrogen, Shanghai, China) with 35S promoter (CaMV35S) [29], followed by being interposed into GV3101 Agrobacterium strain. Agrobacterium strain was transferred into *Arabidopsis thaliana* by floral dip method [30] and independent transgenic *Arabidopsis* lines were developed and verified by PCR amplification. The T_2_ homologous (overexpressing sugarcane *MYB* gene) lines were selected for gene expression analysis, total amino acid contents, and antioxidant capacity analysis whereas for control WT *Arabidopsis* plants were used.

Multiple Expectation Maximization for Motif Elicitation (MEME) platform 4.0 version was used for conserved motifs analysis (http://meme-suite.org/tools/meme, accessed on 8 January 2021) with the following conditions: motif width >6 to <200; maximum motifs 25, as defined before [31]. The TBtools and MEGA7 software was used for phylogenetic analysis, and Maximum Likelihood analysis, respectively [32].

### 2.7. Total Amino Acid and Organic Acid Contents and Their Antioxidant Capacity Assays

For total amino acid contents, the ninhydrin (dissolved in ethanol) procedure was used as described previously [33]. The absorbance of rind samples was collected at 570 nm spectrophotometer (UV-1800). For antioxidant capacity assays, the ferric reducing antioxidant power assay (FRAP) [34], 2,2-diphenyl-1-picrylhydrazyl (DPPH) method [35], and potassium ferricyanide reducing antioxidant power (PFRAP) technique was used as described previously [36]. The absorbance values were taken at 700 nm, 517 nm, and 700 nm, respectively on spectrophotometer UV-1800. In all antioxidant capacity assays, the Trolox standard curve was used. For total organic acid content, the titration method was adopted and the following formula was used to calculate the total organic acid contents as described before [37].

Acid contents in mekv/g of fresh rind (X) = a × T × 200 × 10/n × 50

Whereas the T = 0.1 mol/L of NaOH standard solution; a = 0.1 mol/L of NaOH (standard solution) used for titration in milliliters; 200 = volume of filtrate in milliliters; 10 = is mekv factor for acids calculation [37], as described before; 50 = filtrate aliquot volume; n = weight of sample (sugarcane rind).

### 2.8. Statistical Analysis

The peak area of each amino acid and the organic acid compound was used and normalized by R-language heatmap package and principal component analysis package by FactoMineR R-package (for multivariate analysis) [27,28]. The antioxidant assay graphs, total contents of amino acids and organic acids, standard error, and correlation analysis were performed by using Excel 2010 program (Microsoft, Redmond, WA, USA). Statistix 8.1 software (Inc., State College, PA, USA) was used for statistical analysis and a student *t*-test was performed to compare the transgenic lines and wild-type plants at * *p* < 0.05; ** *p* < 0.01. Antioxidant assays, total amino- and organic acid contents among six sugarcane varieties were compared by least significant difference test at *p* < 0.05 (a, b, c).

## 3. Results

### 3.1. Diversity of Major Amino Acids and Organic Acids from the Sugarcane Rind

A total of 72 amino acids and 55 organic acid compounds were identified from the rind of the six sugarcane varieties. The CAS number, molecular weight, parent ion Q1 (Dal)/daughter ion Q3 (Dal), etc., of each amino acid and organic acid, are represented in Table S1. Among these, 100 compounds, including organic acids and amino acids, were reported for the first time in sugarcane rind. Among all of the quantified compounds, 13 amino acids and 7 organic acids were abundantly present in the rind of all sugarcane varieties and considered major amino acids and organic acids of sugarcane respectively (Figure S1). These compounds perform numerous functions in conferring tolerance to abiotic and biotic stress in plants [38,39,40].

### 3.2. Hierarchical Cluster Analysis of Metabolites from the Six Sugarcane Varieties

**Figure 1:**
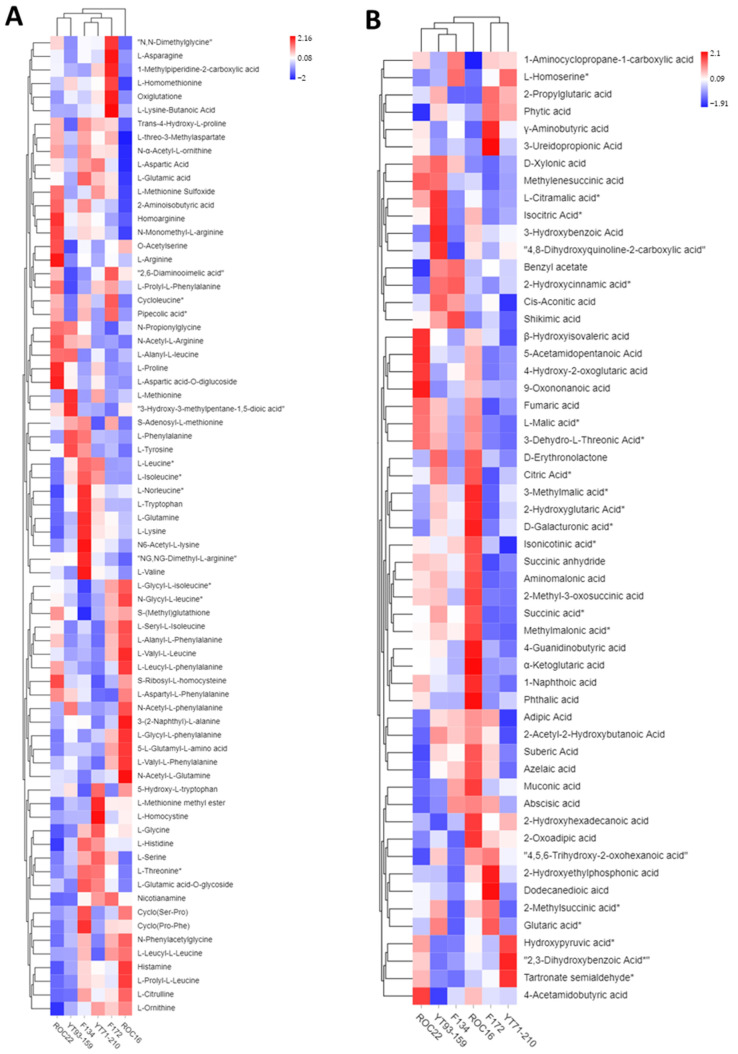
Hierarchical cluster analysis and heat map of amino acid and organic acid compounds in the rind of six sugarcane varieties. (**A**) HCA of amino acid compounds in six sugarcane varieties. (**B**) Diversity of organic acids among six sugarcane varieties. (*) means isotopes. The row signifies the individual compounds (amino acids and organic acids) whereas the column characterizes the six sugarcane varieties. The heat map was generated by the peak area of the individual compound.

Hierarchical cluster analysis (HCA) of amino acid and organic acid compounds are illustrated (Figure 1A,B). The variety-wise amino acid composition HCA showed that the six sugarcane varieties were grouped into two major clusters—ROC16 and the other five varieties, with the latter further divided into three sub-clusters including ROC22 and YT93/159, F172, F134 and YT71/210 (Figure 1A). Among six varieties the ROC16 and F172 were classified into one group independently, not grouped with other varieties, demonstrating their distinctive amino acid characteristics (Figure 1A). The classification of the ROC22 and YT93/159 into the same sub-group and the F134 and YT71/210 into another sub-group, meant that the compositions of the amino acid and organic acid between the two varieties share high similarity (Figure 1A). According to the relative abundance of each amino acid, the amino acid compounds form two major clusters and several sub-groups on HCA (Figure 1A). The concentrations of the typical amino acid such as phenylalanine, tyrosine, and tryptophan were the highest in F134 and YT93/159 (Figure 1A). In addition, the levels of some major sugarcane amino acids such as threonine, valine, glutamine, lysine, leucine, isoleucine, and norleucine were also high in the rind of F134 (Figure 1A). These outcomes suggest that the relative abundance of the amino acids among different sugarcane varieties is diverse.

Moreover, the organic acid HCA again made two distant groups, with F172 and YT71/210 in one group and the other four varieties in another group. The latter group was further divided into three sub-clusters, with ROC22 and ROC16 in one sub-group each and the third sub-group consisting of F134 and YT93/159 (Figure 1B). This result shows that the composition of the organic acid between varieties F172 and YT71/210 is highly similar (Figure 1B), and that this was the same for F134 and YT93/159. The other two subgroups including ROC16 and ROC22 demonstrated their unique organic acid compositions (Figure 1B).

Like the amino acids, according to the abundance of the organic acid compounds, the six varieties of sugarcane were also classified into two major groups and several sub-clusters on HCA (Figure 1B). Generally, the rind of ROC16 showed the highest abundance of the organic acids (Figure 1B), with the levels of succinic acid, methylmalonic acid, citric acid, azelaic acid, and abscisic acid being the highest in ROC16 but the homoserine level being the minimum in ROC16 (Figure 1B). The level of α-ketoglutaric acid was also the highest in ROC16, followed by that of ROC22, and the lowest in F172 and YT71/210 (Figure 1B). The level of shikimic acid was the highest in F134 and YT93/159 (Figure 1A).

### 3.3. Principal Component Analysis (PCA) of Organic Acid and Amino Acid

**Figure 2:**
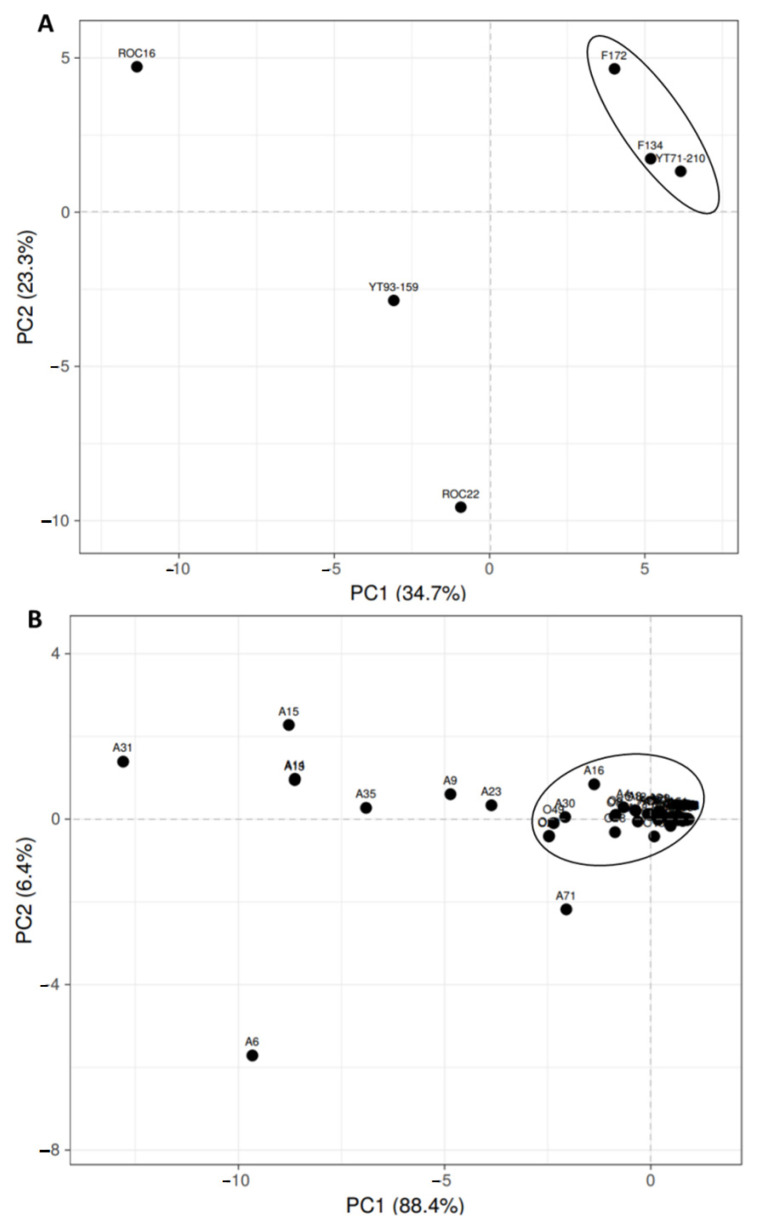
Principal component analysis (PCA) of compounds in six sugarcane varieties. (**A**) Variety-wise scattered PCA, (**B**) individual organic- and amino acid compounds-wise scattered PCA. Here, A means amino acid and O means organic acid compounds and A1–A72 represent the 72 amino acids whereas O1–O55 represent the 55 organic acid compounds; this numbering system corresponds to the serial numbers in Table S1.

The sugarcane variety-wise PCA made one cluster near the intersection point of the x-axis and y-axis (Figure 2A). The F134, YT71/210, and F172 are near to each other and made the same cluster on PCA whereas the ROC16, YT93/159, and ROC22 were scattered and far away from each other (Figure 2A). The PCA of individual organic acid and amino acid in the six varieties showed one large cluster of compounds (in total 119 compounds fall in this cluster) on the intersection point of the x and y axes (Figure 2B). The 8 compounds include A6 (Proline), A9 (Cycloleucine*), A15 (Norleucine*), A19 (1-Methylpiperidine-2-carboxylic acid), A23 (Glutamic acid), A31 (Phenylalanine), A35 (Tyrosine), and A71 (Aspartic acid-O-diglucoside), each of which were scattered and distant from each other (Figure 2B). These compounds contributed a maximum absolute score on both PC1 and PC2 (Figure 2B). The PC1 accounted for 88.4% and PC2 shared 6.4% absolute score values in compound-wise PCA (Figure 2B).

### 3.4. Comprehensive Amino and Organic Acid Biosynthesis Pathways

**Figure 3:**
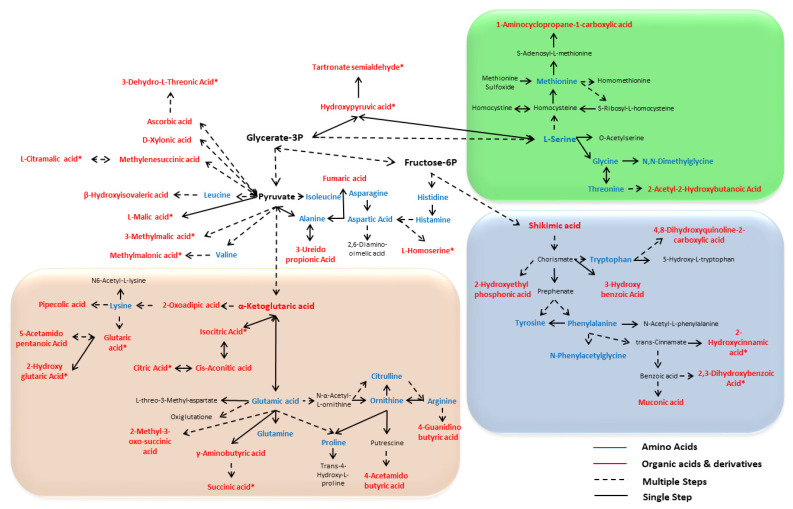
Comprehensive amino acid and organic acid biosynthesis pathway. The blue color compounds represent amino acids whereas the red color compounds represent organic acids that were identified by LC–MS/MS method in sugarcane rinds. All the colored compounds are identified in this study in sugarcane rind whereas the black colored compounds are shown only for describing the sequence and better understanding of metabolic pathway. Dotted lines indicate several enzymatic steps whereas plain lines indicate single steps. The light green, purple, and pink color signify the three major organic acid and amino acid biosynthesis pathways. KEGG plant metabolism (https://www.genome.jp/kegg/pathway.html#metabolism; accessed on 25 November 2021) was used to construct this pathway.

To further understand the biosynthesis pathway of sugarcane amino- and organic acids, we constructed a diagrammatic representation of the comprehensive amino acid and organic acid biosynthesis pathway (Figure 3). The amino acids which were quantified in the rind of the six sugarcane varieties are mostly biosynthesized in glycerate-3P, pyruvate, and fructose-6P pathways (Figure 3). The alanine, serine, leucine, valine, and histidine are key amino acids and are biosynthesized from glycerate-3P, pyruvate, and fructose-6P pathways through single or multiple steps (Figure 3). Some other amino acids and their derivatives are produced with these amino acids, even originating from the organic acid, as they are with the production of glutamic acid from α-ketoglutaric acid (Figure 3). The α-ketoglutaric acid from the TCA cycle is converted into glutamic acid, glutamine, arginine, citrulline, ornithine, and proline (Figure 3).

The levels of α-ketoglutaric acid, citrulline, and ornithine were higher in ROC16 than those in the other five varieties (Figure 1A,B). Chorismate is produced from shikimic acid and is the precursor of the amino acid phenylalanine, tyrosine, and tryptophan (Figure 3). These three kinds of amino acids are the most important amino acids because they give rise to thousands of secondary metabolites. Interestingly, the levels of shikimic acid and these three amino acids were the highest in the F134 variety (Figure 1A,B).

### 3.5. Bioactivity of Amino and Organic Acid in The Rind of Sugarcane

**Figure 4:**
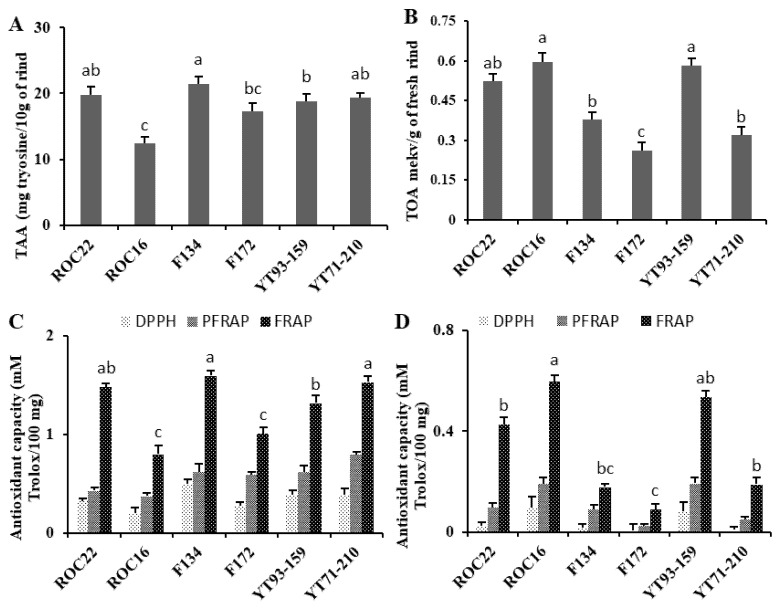
Total contents of amino acid, organic acids and antioxidant capacity assays. (**A**) Total amino acids (TAA) contents, (**B**) total organic acid (TOA) contents, (**C**) antioxidant capacity of amino acids contents, (**D**) antioxidant capacity of organic acid contents. Abbreviations: DPPH: 2,2-diphenyl-1-picrylhydrazyl, FRAP: ferric reducing antioxidant power assay, PFRAP: potassium ferricyanide reducing antioxidant power; Least significant difference was evaluated on each value (*n* = 3 biological replicates) at *p* < 0.05 (a, b, c).

Total contents of amino acids and organic acids were evaluated in the rind of the six sugarcane varieties (Figure 4). The results indicate that F134 (21.46 mg tyrosine equivalents/10 g of rind) has the highest level of total amino acids, followed by ROC22 and YT72/210, whereas ROC16 has the lowest level of total amino acid (Figure 4A). Regarding the total organic acid contents, the ROC16 and YT93/159 represented the maximum, with the content of total organic acids reaching 0.59 and 0.58 mekv per gram of fresh rind, respectively (Figure 4B), while F172 represented the minimum, with a content of total organic acids of 0.26 mekv per gram of fresh rind (Figure 4B). Interestingly, the ROC16 variety displayed maximal content of total organic acid and the minimal content of total amino acids (Figure 4A,B). Our results show that the rind of F134 has the maximum total amino acid content and displays the highest antioxidant activities, followed by YT71/210 and ROC22 (Figure 4A,C). These results show that mutual regulation or balance between the biosynthesis of amino acids and organic acids may exist in the sugarcane variety ROC16, and that this is why the variety has its special characteristics.

As the results of antioxidant capacity assays of amino acids and organic acid in rind extracts from the six varieties illustrate, the values of the FRAP antioxidant capacity assay were maximum, followed subsequently by those of PFRAP and DPPH with the same change direction among the results of the six tested varieties (Figure 4C,D). In the three kinds of assays of the amino acid extracts, the F134 and YT71/210 displayed the maximal antioxidant capacity and the ROC16 represented the minimal antioxidant capacity (Figure 4C). In the three kinds of assays of the organic acid extracts, it was revealed that ROC16 possesses the highest antioxidant capabilities, YT93/159 the second-highest, and varieties F134 and F172 possess the lowest radical-scavenging activity, respectively (Figure 4D).

As the results of the antioxidant assays show, the antioxidant capacity of the amino acids is higher than that of the organic acids. Furthermore, the correlation analysis showed that the total amino acid (TAA) contents have a negative correlation with total organic acid contents, and the TAA content positively and significantly correlates with DPPH and FRAP antioxidant capacity (Table 1), implying that these amino acids in sugarcane largely contributed to the plant’s tolerance to abiotic and biotic stresses.

### 3.6. Transcriptomic Analysis of Genes in Regulating Amino Acid Biosynthesis

**Figure 5:**
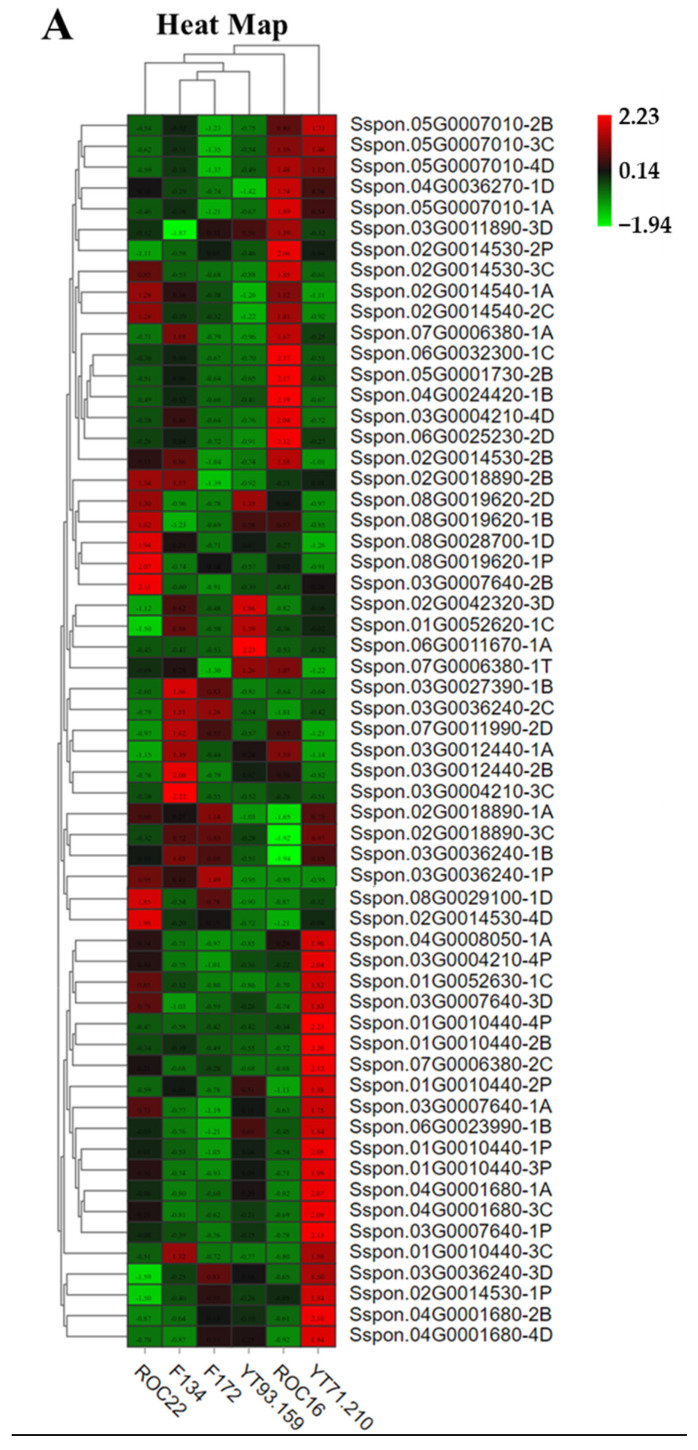

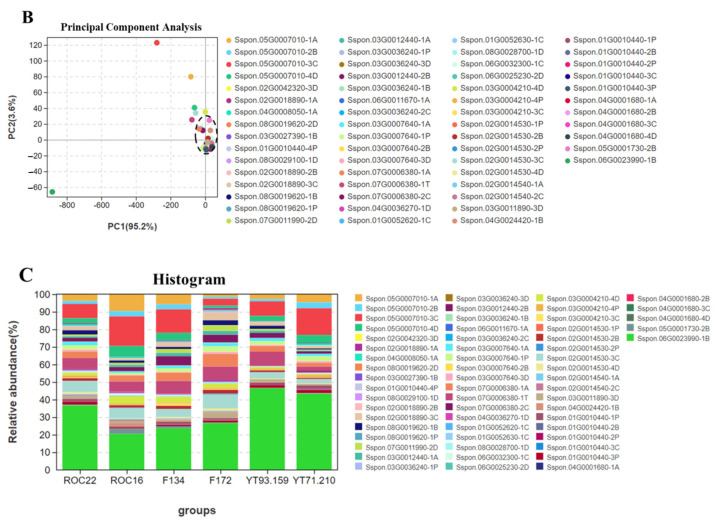
Heat map, principal component analysis, and histogram analysis of transcriptomic data (six sugarcane varieties). (**A**) Heat map of transcriptomic data associated with amino acid biosynthesis genes (the row represents gene expressions and columns signify sugarcane varieties). (**B**) PCA of individual genes distributed on *x*-axis and *y*-axis. (**C**) Histogram analysis of the expression level of the individual gene associated with amino acids biosynthesis in six sugarcane varieties.

To identify the genes involved in the amino acid biosynthesis in rinds of sugarcanes, a hierarchical cluster analysis was performed using the transcriptomic data of amino acid regulatory and biosynthesis genes (Figure 5). The four sugarcane varieties YT93/159, ROC22, YT71/210, and ROC16 did not make any cluster and indicated their uniqueness on HCA whereas the F172 and F134 fell in the same cluster on HCA (Figure 5A). Principal component analysis of transcriptomic data was also performed with the results showing that all genes were grouped near the joining point of the *x* and *y* axes except seven genes (Figure 5B). The PC1 on the *x*-axis presented 95.2% and PC2 on *y*-axis displayed 3.6% absolute score values on the scattered plot (Figure 5B). The seven genes that were scattered on PCA were responsible for maximum absolute score value on both the *x*-axis and *y*-axis PCA. Histogram was achieved by using the expression level of each amino acid biosynthesis gene (Figure 5C).

The seven genes that scattered uniquely in PCA showed high expression in different sugarcane varieties than other genes (Figure 5B). Among these, the *Sspon.06G0023990-1B*, named as *MYB*(*t*) gene, showed distinct characteristics on the PCA and distinctively scattered on PCA (Figure 5B). These results suggest that the candidate *MYB*(*t*) gene is associated with amino acid biosynthesis (Figure 5). Based on our transcriptomic analysis, we cloned the candidate *MYB*(*t*) gene from sugarcane and overexpressed it into the model plant *Arabidopsis*. The raw transcriptomic data is represented in Table S2.

### 3.7. Overexpression of the Candidate MYB(t) Gene Showed a High Accumulation of Amino Acids in Transgenic Arabidopsis

**Figure 6:**
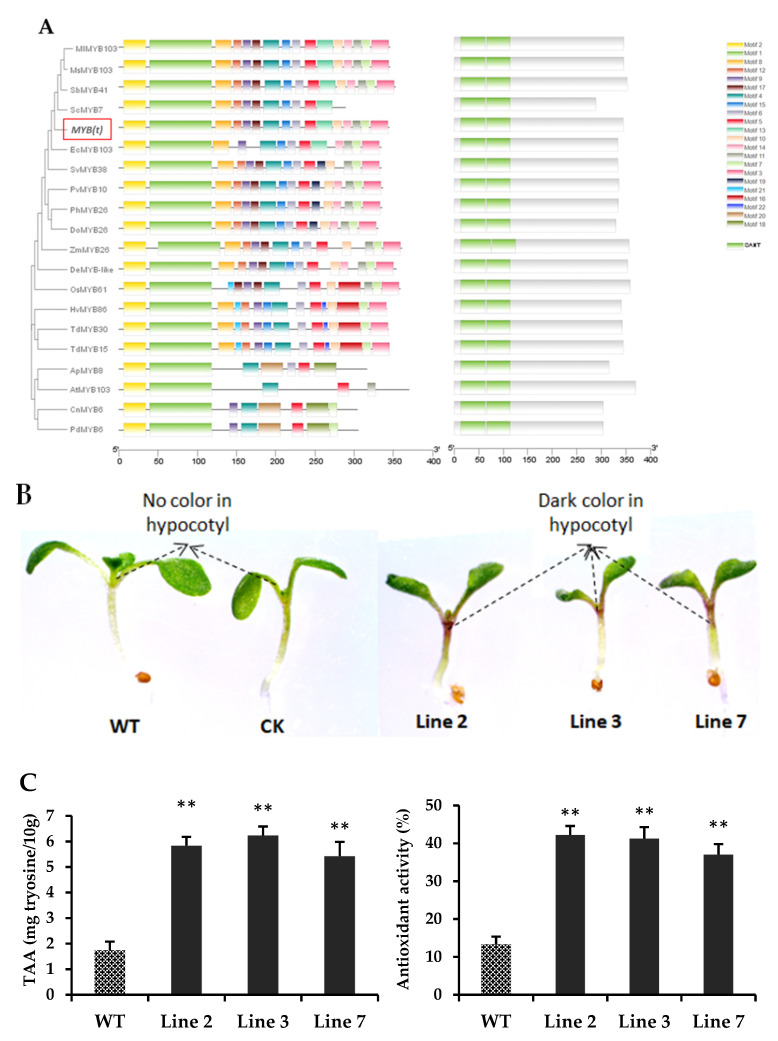

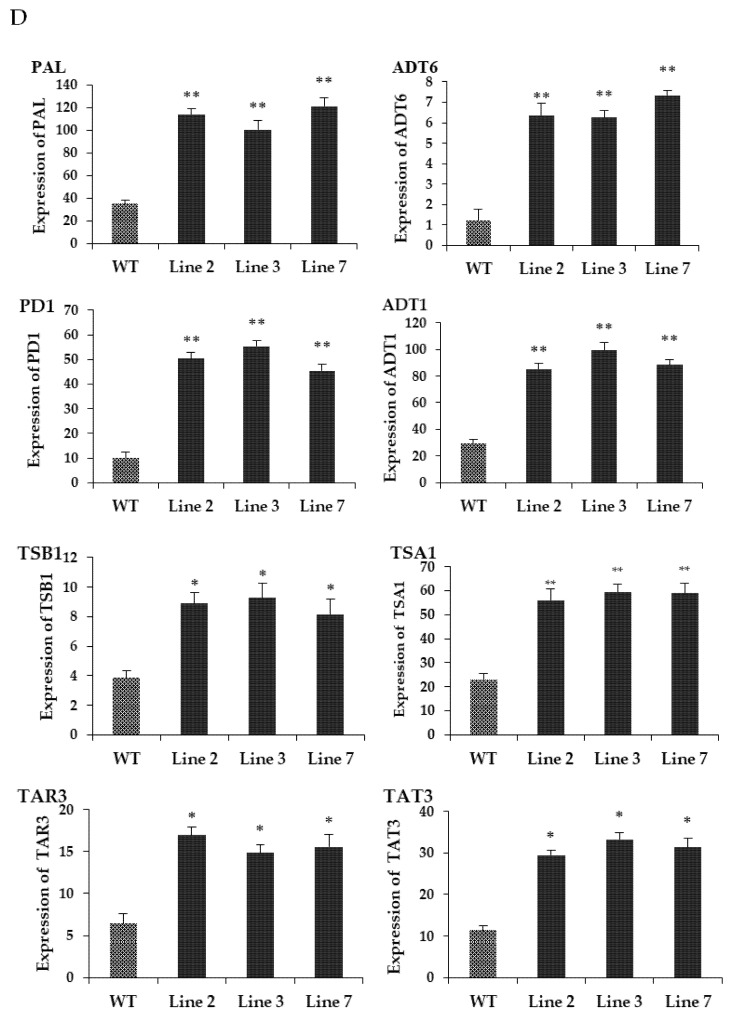
The verification of the *MYB*(*t*) gene in transgenic *Arabidopsis*. (**A**) Phylogenetic, motifs, and domain analysis of *MYB*(*t*) gene with homologous genes from other plant species, highlighting the MEME motif and two conserved SANT domains. (**B**) Comparison of hypocotyls of wild-type and three independent transgenic *Arabidopsis* lines. (**C**) Transgenic plants showed higher amino acid contents and higher antioxidant activity than wild-type plants. (**D**) Expression analysis of amino acid biosynthesis genes in transgenic *Arabidopsis* and wild-type by qRT-PCR. Abbreviations: PAL: phenylalanine ammonia-lyase, ADT6: arogenate dehydratase 6, PD1: prephenate dehydratase 1, ADT1: arogenate dehydratase 1, TSB1: tryptophan synthase beta-subunit 1, TSA1: tryptophan synthase alpha chain, TAR3: tryptophan aminotransferase 3, TAT3: tyrosine aminotransferase 3. The transgenic lines and wild-type plants were compared by students *t*-test at * *p* < 0.05; ** *p* < 0.01. Each graph bar illustrates the mean of three biological repeats.

In this study, the nucleotide sequence of the sugarcane *MYB*(*t*) gene was BLAST in NCBI (https://blast.ncbi.nlm.nih.gov/Blast.cgi?PROGRAM=blastn&PAGE_TYPE=BlastSearch&LINK_LOC=blasthome; assessed on 8 January 2021), as described previously [41], and also in the TAIR *Arabidopsis* website (https://www.arabidopsis.org/Blast/index.jsp; assessed on 5 December 2021). The homologous protein sequence was downloaded, and phylogenetic analysis was performed, the results show that the *ScMYB7* gene (Saccharum cultivar Co 86032) has the highest similarity with *MYB*(*t*) gene (File S1). In *Arabidopsis thaliana*, the *AtMYB103* revealed the highest similarity with the sugarcane *MYB*(*t*) gene (Figure 6A). The *AtMYB103* gene is involved in the biosynthesis of secondary metabolites through activation of the phenylpropanoid pathway [42]. Additionally, two MYB (SANT) domains were identified in the candidate *MYB*(*t*) gene that showed homology with *Sorghum bicolor* (*SbMYB41*) (Figure 6A).

As a result, overexpression of *MYB*(*t*) gene showed a dark purple color phenotype in the hypocotyl and possesses higher amino acid contents, in all transgenic lines, than the wild-type *Arabidopsis* (Figure 6B,C). Moreover, all transgenic lines displayed a higher antioxidant activity than wild-type *Arabidopsis* (Figure 6C).

In addition, all transgenic lines showed high expression levels of genes such as *PAL*, *ADT6*, *PD1*, *ADT1*, *TSB1*, *TSA1*, *TAR3* and *TAT3* involved in the biosynthesis of amino acids (Figure 6D).

These results clearly illustrate that the *MYB*(*t*) gene is involved in amino acid biosynthesis by stimulating the expressions of amino acid biosynthesis genes and significantly enhances the antioxidant activity of all transgenic *Arabidopsis* plants over wild-type plants.

## 4. Discussion

In plants, amino acids play a key role not only in maintaining normal growth development, responding to environmental stresses, adaptability, and survival, but are also precursors of primary and specialized secondary metabolites [18,29,30]. Moreover, plants also use them as a renewable resource for natural products. The shikimate pathway biosynthesizes three critical amino acids, such as tryptophan, tyrosine, and phenylalanine, from which thousands of natural products and hormones are derived [18,43]. Among these three amino acids, the major carbon flux proceeds toward phenylalanine, because more than 9000 compounds are derived from phenylalanine [15]. Among these compounds, lignin (key component of the secondary cell wall) is the most abundant [17]. Volatiles, coumarins, flavonoid compounds, isoflavonoids, anthocyanins, and tannins are some prominent derivatives of phenylalanine [1]. Tyrosine is the precursor to isoquinoline alkaloids, betalains, and quinones, and, in grasses such as sugarcane, they are also responsible for nearly half of total lignin whereas tryptophan is the precursor for indole glucosinolates, auxin, phytoalexins, and alkaloids [16,43]. Therefore, plants sustain their defense, growth and development, and environmental responses, by directing ~30% of photo-synthetic carbon toward amino acid formation [44,45]. The sugarcane rind also contains a substantial proportion of phenolics, flavonoids, alkaloids, anthocyanins, and terpenoids [2,3,32]. However, amino acids and organic acids have gained little attention in cane research, and only a few numbers of these compounds have been reported in sugarcane varieties. In this study, we found that cultivated sugarcane rinds have diverse amino acid and organic acid compounds. Using the LC–MS/MS method, we have identified 72 amino acid and 55 organic acid compounds in the rinds of six cultivated sugarcane varieties, never before reported in previous studies. The rind of F134 has the maximum total amino acid content, while the rinds of ROC16 and YT93/159 have the higher organic acid contents.

Generally, amino- and organic acids play significant roles in modulating plant defense and helping plants to acclimatize to unfavorable stress conditions [8,20,37,38]. In *citrus*, the species tolerant to *Candidatus liberibacter asiaticus bacterium*, possess high levels of plant defense-associated amino acids such as lysine, phenylalanine, tryptophan, asparagine, and tyrosine [46,47]. Our results show that the levels of these amino acids, except asparagine, were the highest in the rind of F134 (Figure 1A). Amino acid derivatives also play a key role in the plant immune system, such as pipecolic acid being derived from lysine which stimulates the camalexin and salicylic acid biosynthesis in *Arabidopsis* leaves [8]. Besides this, amino acids also possess antioxidant activities to assist the plants to detoxify the reactive oxygen species produced under unfavorable environmental conditions [3]. Our results reveal that pipecolic acid level was the highest in F172 whereas F134 had the second-highest level of pipecolic acid and the highest level of lysine (Figure 1). Organic acids are involved in nitrate uptake; malic acid accumulates in the root and assists plants to overcome nitrate deficiency [48]. Organic acid has antioxidant activity, cellular reductant properties, performs multifunctional roles in plant development, growth, and activates plant cellular machinery against abiotic stresses [18]. In plants, organic acid such as α-ketoglutaric acid, shikimic acid, and their derivatives are involved in resistance against pathogens and abiotic stress [18,44]. Our results show that the rind of ROC16 has a high content of α-ketoglutaric acid whereas the level of shikimic acid was high in F134 and YT93/159 (Figure 1B).

In plants, MYB transcription factor genes are involved in biosynthesis of amino acids, phenolics, lignin, flavonoids, anthocyanins, and also play a significant role in plant growth and development [2,43,45,49,50]. In rice, the overexpression of MYB transcription factor gene *OsMYB55* stimulates amino acid metabolism and enhances the total amino acid contents in transgenic rice [49]. Additionally, rice overexpressing the *OsMYB55* also showed tolerance against high temperature due to high amino acid content and improved the growth of transgenic rice plant under high temperature stress [49]. The transgenic rice plants also showed high expression of several genes associated with amino acid metabolism that results in high total amino acid contents [49]. Overexpression of *Petunia hybrida* MYB transcription factor (ODORANT1) gene significantly stimulates the phenylpropanoid metabolism in tomato and enhances the expression of different genes associated with primary and secondary metabolic pathways to accumulate amino acids in transgenic tomato fruit [51]. Heterologous expression of two maize (monocot) transcription factors significantly enhances the flavonoid contents in transgenic tomato (dicot) fruit [52]. Recently, an *MYB* gene from sugarcane has significantly induced the expression of anthocyanins biosynthesis genes and accumulated anthocyanin contents in the hypocotyls of transgenic *Arabidopsis* over the wild-type [2]. In *Arabidopsis*, MYB transcription factors are involved in the biosynthesis of several amino acids [50]. In short, the aforementioned transgenic studies have illustrated that MYB transcription factor significantly regulates the expression of amino acid and phenylpropanoid pathway biosynthesis genes, resulting in the accumulation of amino acids, flavonoids, and anthocyanins. Here, our results demonstrate that ectopic overexpression of sugarcane *MYB*(*t*) significantly enhances the total amino acid contents by stimulating several amino acid biosynthesis genes in transgenic *Arabidopsis* plants (Figure 6D). To our knowledge, none of the transcription factors have been reported to be involved in amino acid metabolism in sugarcane, this is the first study that has illustrated how several major amino acids were quantified and a transcription factor gene *MYB*(*t*) functionally verified to significantly enhance total amino acid content by regulating amino acid biosynthesis genes.

Finally, our study shows that the cultivated sugarcane varieties have diverse level of organic- and amino acid compounds in rind. Sugarcane is the fifth major cultivated crop in the world and is a potential source for essential amino acids. For example, our results show that the rind of F134 has the highest level of essential amino acids such phenylalanine, threonine, valine, tryptophan, lysine, leucine, and isoleucine (Figure 1). Recently, by using functional genomics approaches, reverse genetics and biochemical techniques have been widely used for understanding, regulating, and synthesizing these amino acid pathways in plants [45,53,54]. Additionally, enhancement of amino acid compounds such as tryptophan (a precursor of auxin hormone) is a promising potential strategy for controlling male sterility at high temperatures in *Arabidopsis* [55]. We anticipate that enhancing the tryptophan at the tissue-specific level may also have potential to overcome male sterility in other plant species. Dissimilar level of amino acids in sugarcane germplasms and identification of *MYB* gene (that regulates the amino acid biosynthesis genes to accumulate high amino acid contents) will attract more breeders to regulate amino acid biosynthesis to harvest high amino acid contents which enhance the stress tolerance and nutritive values of cultivated sugarcane crop.

## 5. Conclusions

It was found that cultivated sugarcane rinds have diverse amino acid and organic acid compounds. Using the LC–MS/MS method, we identified 72 amino acid and 55 organic acid compounds in the rinds of six cultivated sugarcane varieties, never before reported in previous studies. The rind of F134 has the maximum total amino acid content, while the rinds of ROC16 and YT93/159 have the higher organic acid contents. The associated study of the sugarcane transcriptome with amino acid contents identified some candidate genes associated with amino acid biosynthesis. Ectopic overexpression of one of the regulatory genes, *MYB*(*t*), showed the enhanced expression of genes involved in amino acid biosynthesis, and the higher amino acid contents in all transgenic *Arabidopsis* plants over the wild-type plants. Thus, identification of organic- and amino acid compounds and functional verification of the associated genes will open a door for further studies of the metabolomics in sugarcane.

## Figures and Tables

**Table 1:** Correlation analysis of total amino acid, organic acid contents and their antioxidant capacity assay. (Bold values indicate the significantly difference at *p* < 0.01).

Variables	Total Amino Acid	Total Organic Acid	DPPH	PFRAP	FRAP
**Total amino acid**	**1.00**				
**Total organic acid**	−0.37	**1.00**			
**DPPH**	**0.90**	−0.28	**1.00**		
**PFRAP**	0.57	−0.64	0.63	**1.00**	
**FRAP**	**0.94**	−0.24	**0.88**	0.56	**1.00**

## Data Availability

All the data is available in the main text.

## Supplementary Materials

The following supporting information can be downloaded at: https://www.mdpi.com/article/10.3390/antiox11071319/s1, Figure S1: Chemical structure of major amino- and organic acids in sugarcane. Table S1: Details of amino acid and organic acid quantified in this study. Table S2: Represents the raw transcriptomic data used in this study. Table S3: Represents the LC–MS/MS data and primer sequences used in this study. File S1: Represents the protein sequences used for constructing phylogenetic tree.
