# Supplementary material for: Transcriptomic and Widely Targeted Metabolomic Approach Identified Diverse Group of Bioactive Compounds, Antiradical Activities, and Their Associated Genes in Six Sugarcane Varieties
Source: Antioxidants (Basel). 2022 Jul 4;11(7):1319. doi: 10.3390/antiox11071319 (PMC9311902; doi:10.3390/antiox11071319)
Supplement: Supplementary file 1 [file antioxidants-11-01319-s001.zip › Supplementary Figures.pdf]

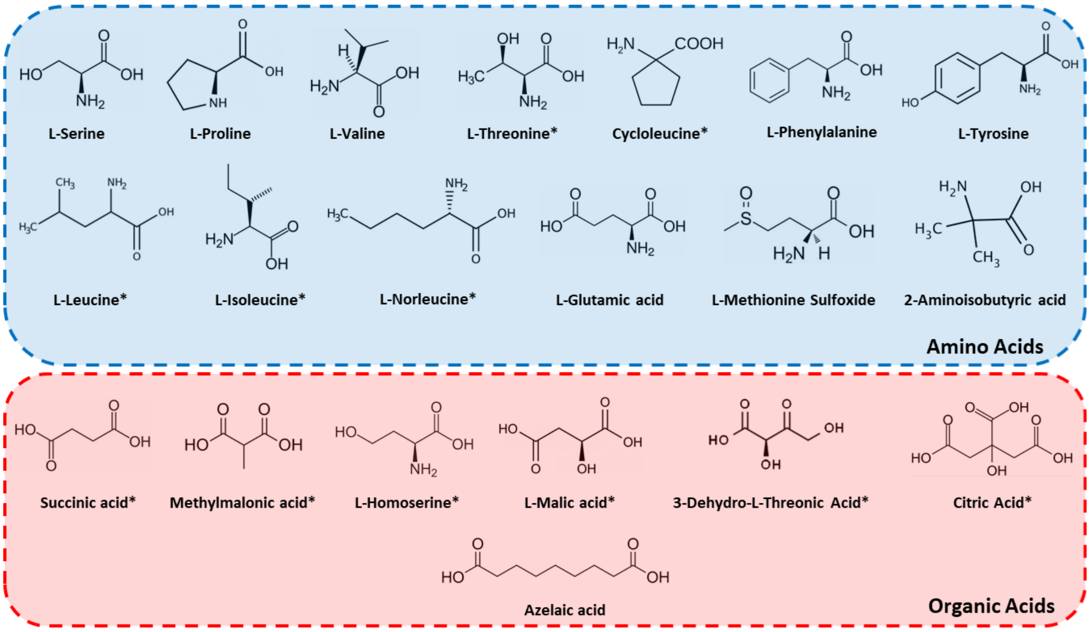

Figure S1 Chemical structure of major amino acids and organic acid in sugarcane.

Internal standard, quality control, and mass spectrometry details:

We have performed a wide-target, high-throughput test, using a relatively quantitative method, we have used internal standard for quality control, adding internal standard in the extraction agent, the purpose is: to calibrate and eliminate the impact of fluctuations in operating conditions on the analysis results, to improve the accuracy of the analysis results. According to the detection of internal standard substances in the sample, such as whether the peak, whether the fluctuation range is too large, so as to judge whether the detection results of metabolites in the sample is stable; About the quality control standard: CV value is the Coefficient of Variation, which is the ratio between the standard deviation of the original data and the mean of the original data, and can reflect the degree of data dispersion. The Empirical Cumulative Distribution Function (ECDF) can be used to analyze the occurrence frequency of CV of substances smaller than the reference value. The higher the proportion of substances with lower CV value in QC samples, the more stable the experimental data.

Every step in the project testing process is equipped with quality control procedures to ensure the stability of experimental operation and testing instrument. Our testing instrument is stable, and the quality control standard is reflected by the CV value of internal standard. The CV value of internal standard data of all samples of the project is in full compliance with the delivery standard. In the delivery data of the project, materials with CV value below 30% of mix samples account for more than 95%, which is in line with the standard. Materials with CV value below 50% as stipulated by the company are deliverable

Under internal standard CV value:

Mws0923 8.645759%, Internal standard information.

High resolution mass spectrometry AB SCIEX 6600 QTOF was used for qualitative detection of mixed samples, which can detect mass to charge ratio to four decimal points to improve qualitative accuracy. Then, AB SciEX 4500 QTRAP was used for quantitative analysis, combining the advantages of untargeted and targeted metabolomics. High resolution mass spectrometry was used for accurate characterization, and triple quadrupole mass spectrometry with high sensitivity, high specificity and excellent quantitative ability was used as a supplementary tool. Matched parameters for metabolite identification include Q1 exact molecular mass, secondary fragmentation, retention time, and isotope.

Table S1 The amino acid and organic acid details quantified in this study.

Serial No.	Amino acid & derivatives Compounds	Formula	Ionization model	Molecular Weight (Da)	CAS	Q1 (Da)	Q3 (Da)
1	Glycine	C2H5NO2	[M+H]+	75.032	56-40-6	76.04	29.99
2	N,N-Dimethylglycine	C4H9NO2	[M+H]+	103.063	1118-68-9	104.07	86
3	2-Aminoisobutyric acid	C4H9NO2	[M+H]+	103.063	62-57-7	104.07	58
4	Serine	C3H7NO3	[M+H]+	105.043	56-45-1	106	60
5	Histamine	C5H9N3	[M+H]+	111.08	51-45-6	112.09	95
6	Proline	C5H9NO2	[M+H]+	115.063	147-85-3	116.07	70.1
7	Valine	C5H11NO2	[M+H]+	117.079	72-18-4	118.09	72.1
8	Threonine*	C4H9NO3	[M+H]+	119.058	72-19-5	120.07	74
9	Cycloleucine*	C6H11NO2	[M+H]+	129.079	52-52-8	130.09	84.08
10	Pipecolic acid*	C6H11NO2	[M+H]+	129.079	4043-87-2	130.09	84.08
11	N-Propionylglycine	C5H9NO3	[M-H]-	131.058	21709-90-0	130.05	86
12	Trans-4-Hydroxy-proline	C5H9NO3	[M+H]+	131.058	51-35-4	132.07	86
13	Leucine*	C6H13NO2	[M+H]+	131.095	61-90-5	132.1	86.2
14	Isoleucine*	C6H13NO2	[M+H]+	131.095	73-32-5	132.1	86
15	Norleucine*	C6H13NO2	[M+H]+	131.095	327-57-1	132.1	86
16	Asparagine	C4H8N2O3	[M+H]+	132.054	70-47-3	133.06	74
17	Ornithine	C5H12N2O2	[M+H]+	132.09	70-26-8	133	116
18	Aspartic Acid	C4H7NO4	[M-H]-	133.038	56-84-8	132.03	88
19	1-Methylpiperidine-2-carboxylic acid	C7H13NO2	[M+H]+	143.095	7730-87-2	144.1	84.08
20	Glutamine	C5H10N2O3	[M+H]+	146.069	56-85-9	147.08	84
21	Lysine	C6H14N2O2	[M+H]+	146.106	56-87-1	147.11	84
22	O-Acetylserine	C5H9NO4	[M+H]+	147.053	5147-00-2	148.06	88
23	Glutamic acid	C5H9NO4	[M+H]+	147.053	56-86-0	148.06	84
24	threo-3-Methylaspartate	C5H9NO4	[M-H]-	147.053	6061-13-8	146.05	102.06
25	Methionine	C5H11NO2S	[M+H]+	149.051	63-68-3	150.06	61
26	Histidine	C6H9N3O2	[M+H]+	155.069	71-00-1	156.08	110
27	3-Hydroxy-3-methylpentane-1,5-dioic acid	C6H10O5	[M-H]-	162.053	503-49-1	161.05	99
28	Homomethionine	C6H13NO2S	[M+H]+	163.067	25148-30-5	164.07	56.05
29	Methionine methyl ester	C6H13NO2S	[M+H]+	163.067	10332-17-9	164.07	104
30	Methionine Sulfoxide	C5H11NO3S	[M+H]+	165.046	3226-65-1	166.05	102
31	Phenylalanine	C9H11NO2	[M+H]+	165.07	63-91-2	166.09	120.08
32	N-α-Acetyl-ornithine	C7H14N2O3	[M-H]-	174.1	6205-08-9	173.11	131.08

Arginine	C6H14N4O2	[M+H]+	174.112	74-79-3	175.12	116
Citrulline	C6H13N3O3	[M+H]+	175.096	372-75-8	176.1	113
Tyrosine	C9H11NO3	[M+H]+	181.074	60-18-4	182.08	136.1
Cyclo(Ser-Pro)	C8H12N2O3	[M+H]+	184.086	-	185.09	70.06
N-Acetyl-Glutamine	C7H12N2O4	[M-H]-	188.08	2490-97-3	187.07	125
Glycyl-isoleucine*	C8H16N2O3	[M+H]+	188.116	19461-38-2	189.12	86.1
N-Glycyl-leucine*	C8H16N2O3	[M+H]+	188.116	869-19-2	189.12	86
N6-Acetyl-lysine	C8H16N2O3	[M+H]+	188.116	692-04-6	189.12	126
Homoarginine	C7H16N4O2	[M+H]+	188.127	156-86-5	189.13	144
N-Monomethyl-arginine	C7H16N4O2	[M+H]+	188.127	17035-90-4	189.13	70.07
2,6-Diaminooimelic acid	C7H14N2O4	[M+H]+	190.095	583-93-7	191.1	128
N-Phenylacetylglycine	C10H11NO3	[M+H]+	193.074	500-98-1	194.08	91
Alanyl-leucine	C9H18N2O3	[M+H]+	202.132	3303-34-2	203.14	86.1
NG,NG-Dimethyl-arginine	C8H18N4O2	[M+H]+	202.143	30315-93-6	203.15	70.07
Tryptophan	C11H12N2O2	[M-H]-	204.09	73-22-3	203.08	116.05
N-Acetyl-phenylalanine	C11H13NO3	[M-H]-	207.09	2018-61-3	206.08	58.03
3-(2-Naphthyl)-L-alanine	C13H13NO2	[M-H]-	215.095	58438-03-2	214.1	134
N-Acetyl-Arginine	C8H16N4O3	[M+H]+	216.122	155-84-0	217.13	158
5-Glutamyl-amino acid	C8H14N2O5	[M-H]-	218.09	5875-41-2	217.08	199
Seryl-Isoleucine	C9H18N2O4	[M+H]+	218.127	91086-51-0	219.13	60.04
5-Hydroxy-tryptophan	C11H12N2O3	[M+H]+	220.085	56-69-9	221.09	204
Glycyl-phenylalanine	C11H14N2O3	[M+H]+	222.1	721-66-4	223.11	120.09
Prolyl-Leucine	C11H20N2O3	[M+H]+	229.154	52899-07-7	229.15	70.07
Valyl-Leucine	C11H22N2O3	[M+H]+	231.17	3989-97-7	231.16	72.08
Lysine-Butanoic Acid	C10H22N2O4	[M+H]+	234.158	80407-71-2	235.1	118.3
Alanyl-Phenylalanine	C12H16N2O3	[M+H]+	236.116	3061-90-3	237.12	120.09
Cyclo(Pro-Phe)	C14H16N2O2	[M+H]+	245.128	3705-26-8	245.12	120.08
Leucyl-Leucine	C12H24N2O3	[M+H]+	245.186	3303-31-9	245.18	86.1
Prolyl-Phenylalanine	C14H18N2O3	[M+H]+	263.139	13589-02-1	263.13	70.07
Valyl-Phenylalanine	C14H20N2O3	[M+H]+	264.147	3918-92-1	265.15	72.08
S-Ribosyl-homocysteine	C9H17NO6S	[M-H]-	267.078	-	266.09	134.05
Homocystine	C8H16N2O4S2	[M+H]+	268.055	626-72-2	269.06	136
Leucyl-phenylalanine	C15H22N2O3	[M+H]+	278.163	56217-82-4	279.17	120.08
Aspartyl-Phenylalanine	C13H16N2O5	[M+H]+	280.106	13433-09-5	281.11	166
Nicotianamine	C12H21N3O6	[M+H]+	303.143	34441-14-0	304.15	185.09
Glutamic acid-O-glycoside	C11H19NO9	[M-H]-	309.106	-	308.1	146.1
S-(Methyl)glutathione	C11H19N3O6S	[M+H]+	321.099	2922-56-7	322.11	130
S-Adenosyl-methionine	C15H22N6O5S	[M+H]+	398.137	29908-03-0	399.2	250
Aspartic acid-O-diglucoside	C16H27NO14	[M+H]+	457.143	-	458.1	116.3
Oxiglutatione	C20H32N6O12S2	[M-H]-	612.152	27025-41-8	611.14	306.08
Succinic anhydride						
1-Aminocyclopropane-1-carboxylic acid	C4H4O3	[M+H]+	100.018	108-30-5	101.02	55.02
γ-Aminobutyric acid	C4H7NO2	[M+H]+	101.048	22059-21-8	102.05	56.05
Hydroxypyruvic acid*	C4H9NO2	[M+H]+	103.063	56-12-2	104.07	68.8
Tartronate semialdehyde*	C3H4O4	[M-H]-	104.011	1113-60-6	103	59.01
Fumaric acid	C3H4O4	[M-H]-	104.011	2480-77-5	103	59.01
Erythronolactone	C4H4O4	[M-H]-	116.011	110-17-8	115	71
Succinic acid*	C4H6O4	[M-H]-	118.027	15667-21-7	117	59

80	Methylmalonic acid*	C4H6O4	[M-H]-	118.027	110-15-6	117.02	73
81	β-Hydroxyisovaleric acid	C4H6O4	[M-H]-	118.027	516-05-2	117.02	73
82	Aminomalonic acid	C5H10O3	[M-H]-	118.063	625-08-1	117.06	59
83	Homoserine*	C3H5NO4	[M-H]-	119.022	1068-84-4	118	74
84	Isonicotinic acid*	C4H9NO3	[M+H]+	119.058	672-15-1	120.07	74.1
85	2-Hydroxyethylphosphonic acid	C6H5NO2	[M+H]+	123.032	55-22-1	124.04	78.03
86	Methylenesuccinic acid	C2H7O4P	[M-H]-	126.008	22987-21-9	125	78.96
87	2-Methylsuccinic acid*	C5H6O4	[M-H]-	130.027	97-65-4	129.02	85.03
88	Glutaric acid*	C5H8O4	[M-H]-	132.042	498-21-5	131.03	87.05
89	3-Ureidopropionic Acid	C5H8O4	[M-H]-	132.042	110-94-1	131.03	87.05
90	Malic acid*	C4H8N2O3	[M-H]-	132.053	462-88-4	131.05	70.03
91	3-Dehydro-L-Threonic Acid*	C4H6O5	[M-H]-	134.022	97-67-6	133.01	71.01
92	3-Hydroxybenzoic Acid	C4H6O5	[M-H]-	134.022	-	133.01	71.01
93	Muconic acid	C7H6O3	[M-H]-	138.032	99-06-9	137.02	93.03
94	4-Acetamidobutyric acid	C6H6O4	[M-H]-	142.027	1119-72-8	141.02	59
95	4-Guanidinobutyric acid	C6H11NO3	[M+H]+	145.074	3025-96-5	146.08	86
96	2-Methyl-3-oxosuccinic acid	C5H11N3O2	[M+H]+	145.085	463-00-3	146.09	87
97	α-Ketoglutaric acid	C5H6O5	[M-H]-	146.022	-	145.01	57.03
98	Adipic Acid	C5H6O5	[M-H]-	146.022	328-50-7	145.01	101
99	2-Acetyl-2-Hydroxybutanoic Acid	C6H10O4	[M-H]-	146.058	124-04-9	145.05	101
100	3-Methylmalic acid*	C6H10O4	[M-H]-	146.058	-	145.05	83.05
101	Citramalic acid*	C5H8O5	[M-H]-	148.037	152204-30-3	147.03	57.03
102	2-Hydroxyglutaric Acid*	C5H8O5	[M-H]-	148.037	6236-09-5	147.03	87.1
103	Benzyl acetate	C5H8O5	[M-H]-	148.037	13095-48-2	147.03	57.03
104	2,3-Dihydroxybenzoic Acid*	C9H10O2	[M-H]-	150.068	140-11-4	149.06	74.9
105	5-Acetamidopentanoic Acid	C7H6O4	[M-H]-	154.027	303-38-8	153.02	109.03
106	2-Oxoadipic acid	C7H13NO3	[M-H]-	159.09	1072-10-2	158.08	116.07
107	4-Hydroxy-2-oxoglutaric acid	C6H8O5	[M-H]-	160.037	3184-35-8	159.1	87
108	2-Hydroxycinnamic acid*	C5H6O6	[M-H]-	162.016	1187-99-1	161	73
109	Phthalic acid	C9H8O3	[M-H]-	164.047	583-17-5	163.04	119.05
110	Xylonic acid	C8H6O4	[M-H]-	166.027	88-99-3	165.02	77.04
111	1-Naphthoic acid	C5H10O6	[M-H]-	166.048	526-91-0	165.04	75
112	9-Oxononanoic acid	C11H8O2	[M-H]-	172.052	86-55-5	171.04	127.05
113	Cis-Aconitic acid	C9H16O3	[M-H]-	172.11	2553-17-5	171.1	99.08
114	Shikimic acid	C6H6O6	[M-H]-	174.016	585-84-2	173.02	137
115	2-Propylglutaric acid	C7H10O5	[M-H]-	174.053	138-59-0	173.05	93
116	Suberic Acid	C8H14O4	[M-H]-	174.089	32806-62-5	173.08	59.01
117	4,5,6-Trihydroxy-2-oxohexanoic acid	C8H14O4	[M-H]-	174.089	505-48-6	173.08	111
118	Azelaic acid	C6H10O6	[M-H]-	178.048	56742-44-0	177.04	113.02
119	Isocitric Acid*	C9H16O4	[M-H]-	188.105	123-99-9	187.1	125
120	Citric Acid*	C6H8O7	[M-H]-	192.027	320-77-4	191.02	111.01
121	Galacturonic acid*	C6H8O7	[M-H]-	192.027	77-92-9	191.02	111.01
122	4,8-Dihydroxyquinoline-2-carboxylic acid	C6H10O7	[M-H]-	194.033	685-73-4	193.04	72.8
123	Dodecanedioic aicd	C10H7NO4	[M-H]-	205.038	59-00-7	204.03	160.04
124	Abscisic acid	C12H22O4	[M-H]-	230.152	693-23-2	229.14	211

125	2-Hydroxyhexadecanoic acid	C15H20O4	[M-H]-	264.136	21293-29-8	263.13	204.12
126	Phytic acid	C16H32O3	[M-H]-	272.235	764-67-0	271.23	225.22
127	Glycine	C6H18O24P6	[M-H]-	659.861	83-86-3	658.85	482.93

*means isomers
